# Anæsthesia

**Published:** 1890-10

**Authors:** William H. Burr

**Affiliations:** Wilmington, Del.


### Anæsthesia.

BY WILLIAM H. BURR, M.D., WILMINGTON, DEL.

If it is possible, on any medical question, to bring order out of confusion, it would seem as though the above was one of vital importance. It is not as to which is the safer, ether or chloroform, but how should they be administered. A good deal of very positive opinion has been expressed, the best that I have seen being several monographs by Dr. Julian J. Chisolm, of Baltimore, which should be generally read. In the June number of the *University Medical Magazine* appears a very able article by Dr. J. T. Carpenter, which refers to the directions given by the London Hospital Surgeons many years ago, in which it is urged that atmospheric air must be freely admitted with the anæsthetic; that the inhalation be occasionally suspended, and that the patient be slowly brought under the influence of the drug, and finally, that in many cases full anæsthesia should not be pushed. Dr. Carpenter admits that these directions have been followed the world over, and then declares them to be all erroneous, and to arise from false conceptions of the proper nature of the anæsthetic action. He says, first, that an anæsthetic should be given with the least admixture of air so as to get the maximum effect from a minimum dose; that one should not administer the poison chloroform with its antidote air, and gives as a reason that you will surely deluge the vital centres with an undue amount of poison.

I can not see the philosophy of this. I think all are agreed that the last clause of the London Hospital directions should be discarded, namely, "In many cases full anæsthesia should not be pushed." But in regard to the other directions, which Dr. Carpenter says are followed over the whole world, I think they are in accord with physiology and common sense. Dr. Carpenter has had a happy experience of thirty years, and that counts for a good deal, but it can not be possible that he has personally administered chloroform all that time. And in the matter of anæsthesia, my experience is that the administrator follows his own ideas and experience, and takes the responsibility himself. If he does not, he ought to, for each one should administer anæsthetics according to his own ideas and those of nobody else.

I have had experience with both methods of administration, the gradual and forced. With the latter I have seen men and women struggling as if in death agony, and requiring half a dozen assistants to hold them down, praying and singing and telling all the little secrets of their lives. The former method I always use, and have never had a patient excited since I began. My theory in using gradual and careful administration is, that the more slowly the nerve centres and respiratory organs are brought under the influence of anæsthetics the less danger of their being overwhelmed by them. This is somewhat on the same principle as that one accustomed to taking alcoholic beverages can take at one time what would kill any one not accustomed to it. It is not supposed for one instant the absorption is much less in the one case than the other, but the nerve centres of the toper had become inured to a large dose and his elimination is probably also increased.

Every one holds his breath with anxiety while a patient is going through with the primary stages of anæsthesia. There is gastric disturbance, irritation of the respiratory organs, irregular breathing, and finally, just before complete anæsthesia, that horrible condition between sleeping and waking in which the patient apparently ceases to breathe at all, and the practitioner does not know whether he is going to breathe again or not. Then when regular breathing comes with complete anæsthesia there is relief to all concerned. To me the longer the anæsthesia proceeds properly, within certain limits, the safer I feel.

Lyman asserts that ether kills by a mechanical surfeit, that it is capable of uniting with the protoplasm in a moving equilibrium if the quantity is not too large, but by an excessive amount it causes death in the same way as smoke or drowning, by interfering with the oxygenation; and that chloroform belonging, as it does, to the chlorine, bromine, and iodine series, can only be associated with the elements of living matter in a stable equilibrium and therefore is a chemical poison.

Whether they are mechanical or chemical poisons, or both, it would seem, if their action is at all analogous to that of other poisons, to flood the tissues suddenly with a pure vapor, excluding air, would overwhelm the protoplasm and cause death.—*Medical and Surgical Reporter*.

